# Geochemical provenance of an Indo-Arabian stone anchor from Manikapatna highlights the medieval maritime trade of India

**DOI:** 10.1038/s41598-022-17910-9

**Published:** 2022-08-09

**Authors:** Sila Tripati, Jyotiranjan S. Ray, Rudra Prasad Behera, Prakash Babu, Milan Kumar Mahala, Murali Kocherla, Vijay Khedekar

**Affiliations:** 1 CSIR-National Institute of Oceanography, Goa, 403004 India; 2 National Centre for Earth Science Studies, Akkulam, Thiruvananthapuram, 695011 India; 3 Physical Research Laboratory, Navrangpura, Ahmedabad 380009 India; 4 Deptartment of Archaeology, Government of Odisha, Bhubaneswar, 751014 India

**Keywords:** Environmental social sciences, Ocean sciences, Solid Earth sciences

## Abstract

India is one of the oldest maritime nations in the world, and the overseas contacts date back to the third millennium BCE. Besides several archaeological vestiges, numerous stone anchors of various types have been documented during maritime archaeological explorations along the Indian littoral. During a recent maritime archaeological exploration, a broken Indo-Arabian stone anchor, of the Medieval period, was discovered along the Manikapatna coast of Odisha, Indian eastern littoral. In an attempt to determine the provenance of the anchor, we carried out a detailed petrographic, geochemical (major/trace elements) and Sr–Nd isotopic investigation. The results of our study reveal that the stone of the anchor had been cut out of a geologically young, vesicular, subalkalic basalt lava flow. Source fingerprinting done using petrographic, geochemical and isotopic data, suggests that contrary to the general perception, the anchor rock did not come from any local rock formations. All data point to the most likely scenario that the anchor rock was sourced from one of the lava flows of the Deccan Traps at Palitana in the Saurashtra region of Gujarat, western India. This result confirms the existence of Medieval maritime trading between western and eastern Indian states.

## Introduction

**Figure 1:**
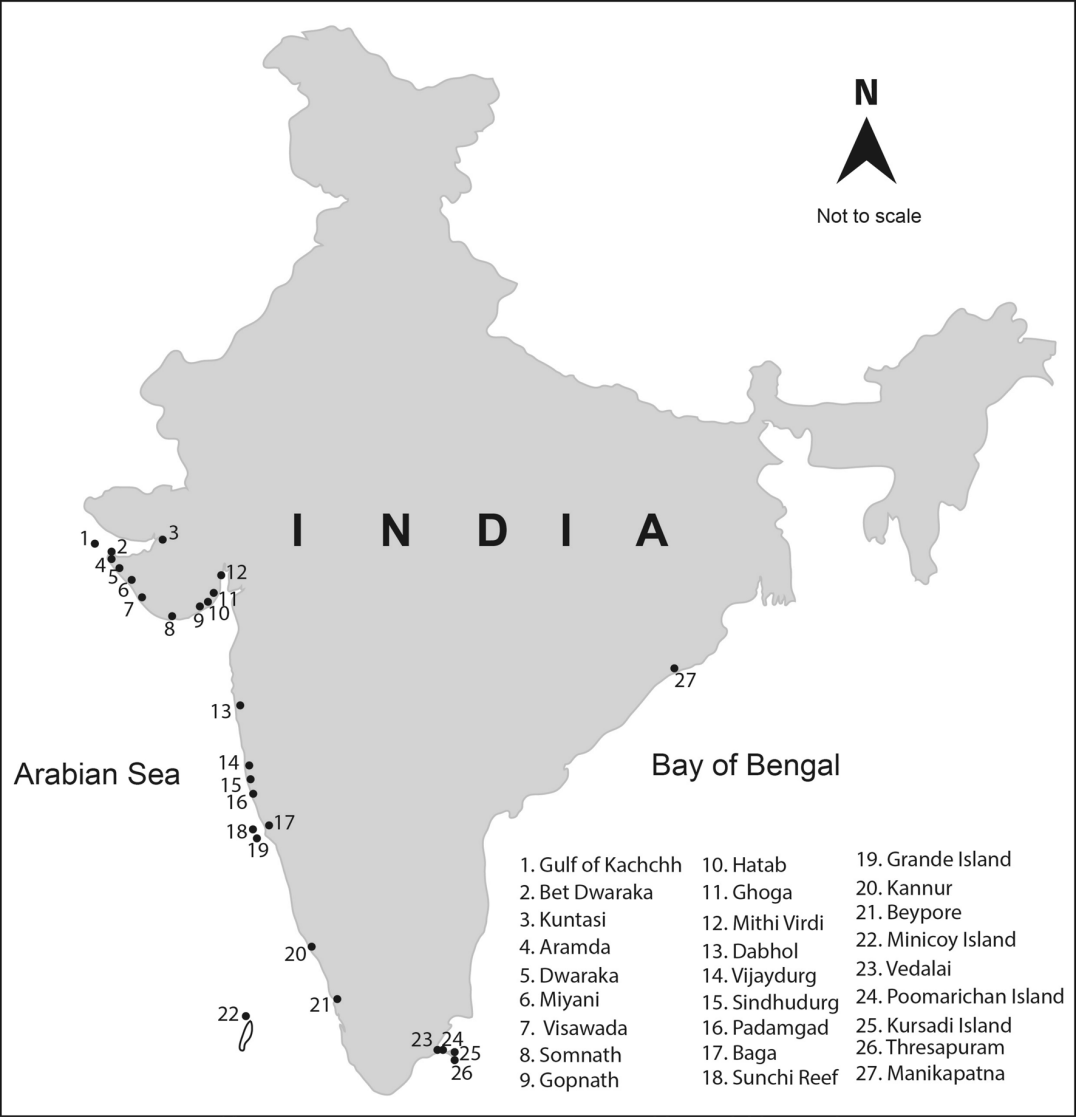
Stone Anchor sites of India.

India is one of the oldest maritime nations in the world with its trading history dating back to the third millennium BCE. Archaeological findings of cargo, crafts, anchors, timber, etc. provide insight into India’s past maritime contacts with other countries/civilizations. The use of anchors by boats and ships engaged in such maritime activity has also been recorded. The earliest anchors were made up of large stones, which were tied with ropes and lowered to hold the ground in the sea/river/lake. Subsequent records show diversification of stone anchors and the use of anchors made up of wood, lead and iron. The Harappans were the earliest mariners from the Indian subcontinent^1,2^ and they had used stone anchors, evidence for this comes from Lothal^3^ and Kuntasi^4^, along the Gujarat coast, western India. A wide range of stone anchors, particularly composite, Indo-Arabian, ring stone and single hole types, have been recorded during maritime archaeological explorations along the Indian coast^5^ (Fig. 1).

The findings of these stone anchors suggest the existence of maritime contacts between various coastal kingdoms/states of the Indian subcontinent during different periods of history (Table 1). During a recent exploration^6,7^ a broken Indo-Arabian type of stone anchor was discovered along the Manikapatna coast of Chilika, in the eastern Indian state of Odisha. The anchor rock that did not appear to have come from any of the local formations of the coastal Odisha likely holds clues to Medieval maritime trade between Odisha and the place of origin of the anchor. To determine the provenance of the anchor rock, we have carried out a detailed petrographic, geochemical and isotopic investigation. The results of these investigations are presented and their implications for the maritime trade of ancient India, in particular of Odisha, are discussed.

**Table 1:** Details of stone anchors found along the Indian coast.

Name of sites	Composite anchor	Indo-Arabian anchor	Ring stone anchor	Single hole anchor	Killick anchor	Total
*West coast*						
**Gujarat**						
Gulf of Kachchh	–	1	–	–	–	1
Bet Dwarka	13	7	1	–	–	21
Aramda	1	1	–	–	–	2
Dwarka	35	63	24	1	–	123
Miyani	2	6	4	–	–	12
Visawada	10	2	1	1	–	14
Kindar Kheda	1	–	–	–	–	1
Srinagar	1	–	–	–	–	1
Ghumli	–	–	1	–	–	1
Navi Bundar	1	–	–	–	–	1
Somnath	6	2	35	–	–	43
Mul Dwarka (Kodinar)	1	–	–	–	–	1
Gopnath	–	1	–	–	–	1
Hatab	–	4	–	–	–	4
Ghogha	1	18	–	–	–	19
Mithi Virdi	–	4	–	–	–	4
**Maharashtra**						
Dabhol	–	4	–	–	–	4
Vijaydurg	1	23	–	–	–	24
Sindhudurg	3	5	–	–	–	8
Padmagad	–	1	–	–	–	1
Vengurla Rock	–	–	1	–	–	1
**Goa**						
Baga	–	1	–	–	–	1
Sunchi Reef	–	1	1	–	–	2
Grande Island	–	2	–	–	–	2
**Kerala**						
Beypore	–	1	–	–	–	1
Kannur	–	1	–	–	–	1
Kollam	–	–	1	–	–	1
Lakshadweep Island						
Minicoy Island	–	1	–	–	–	1
*East coast*						
**West Bengal**						
Harinarayanpur	–	–	–	1	–	1
**Odisha**						
Astaranga	–	–	–	–	1	1
Belkhandi	–	–	–	1	–	1
Manikapatna	–	1	–	–	–	1
Chilika Lake	4	–	–	1	–	5
** Andhra Pradesh**						
Kottapatnam	–	–	–	1	–	1
**Tamil Nadu**						
Manapad	–	–	–	5	–	5
Kursadi Island	–	1	–	–	–	1
Poomarichan Island	–	1	–	–	–	1
Vedalai	–	1	–	–	–	1
Periapattinam	–	–	–	1	–	1
Threspuram	–	1	–	–	–	1

## Maritime trade and stone anchor of Manikapatna

**Figure 2:**
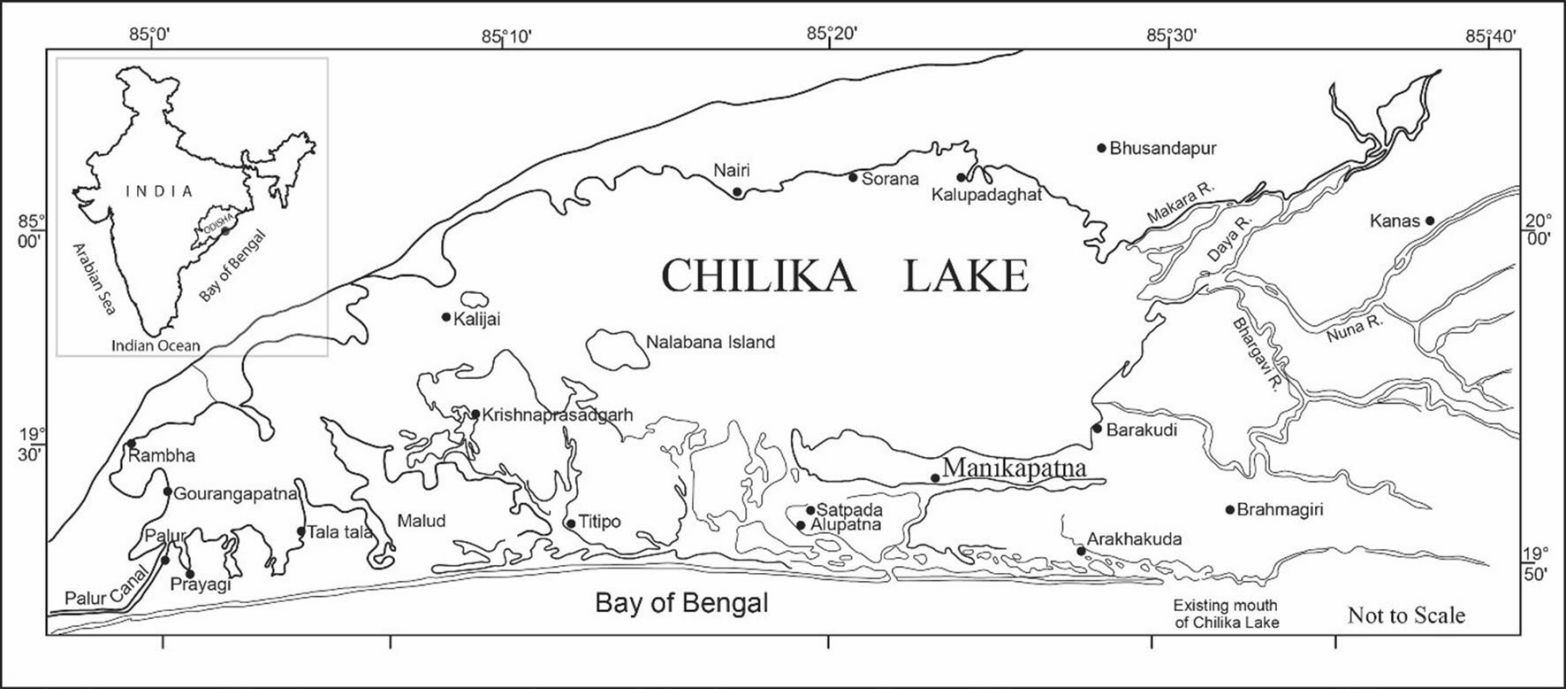
Figure showing the location of Manikapatna and nearby localities around the Chilika Lake, state of Odisha, India.

People of Odisha had ventured into the sea for marine resources as far back as 4000 years ago and were engaged in maritime activities at least since 800 BCE^8^. Several ports and trade centers existed along the Odisha coast and had overseas trade relations at various historical points, some of these ports are explored and excavated, and Manikapatna is noteworthy among them. Manikapatna lies on the bank of Chilika Lake (Fig. 2) and served as a port until late Medieval times. It is believed that Chilika, the biggest brackish water inshore lake connected to the Bay of Bengal, had provided safe passage and shelter to the vessels those voyaged to distant lands. Until 1989, Manikapatna was unknown as a port.

The Odisha Institute of Maritime and Southeast Asian Studies (OIMSEAS), Bhubaneswar carried out the excavations at Manikapatna between 1989 and 1993^9,10^, the site was excavated again in 2010 by the Deccan College, Pune^11^. The discoveries from these excavations are broadly classified into two phases, the first phase is datable from the 2nd century BCE to the 5th/6th century CE, and the second phase lasted from the 9th to 19th century CE. The findings of the excavation include Knobbed ware, Rouletted ware, Khorasthi inscription and coins belonging to the Puri Kushan (1st century CE), Rajaraja Chola (985–1016 CE), Sahassamalla of the Polonnaruva period Sri Lanka and Chinese (14th century CE) as well as Chinese Porcelain (1368–1644 CE) belonging to the Yuan and Ming dynasties^6–9^.

**Figure 3:**
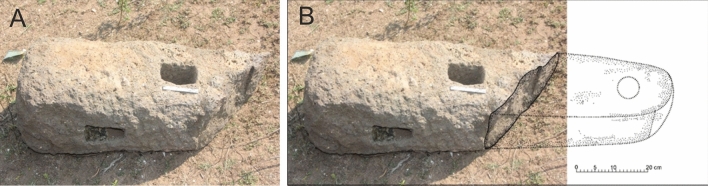
(**A**) Indo-Arabian broken stone anchor found at Manikapatna on Chilika Lake, Odisha; (**B**) Likely outline of the original anchor, reconstructed based on similar anchors found elsewhere^12^.

**Figure 4:**
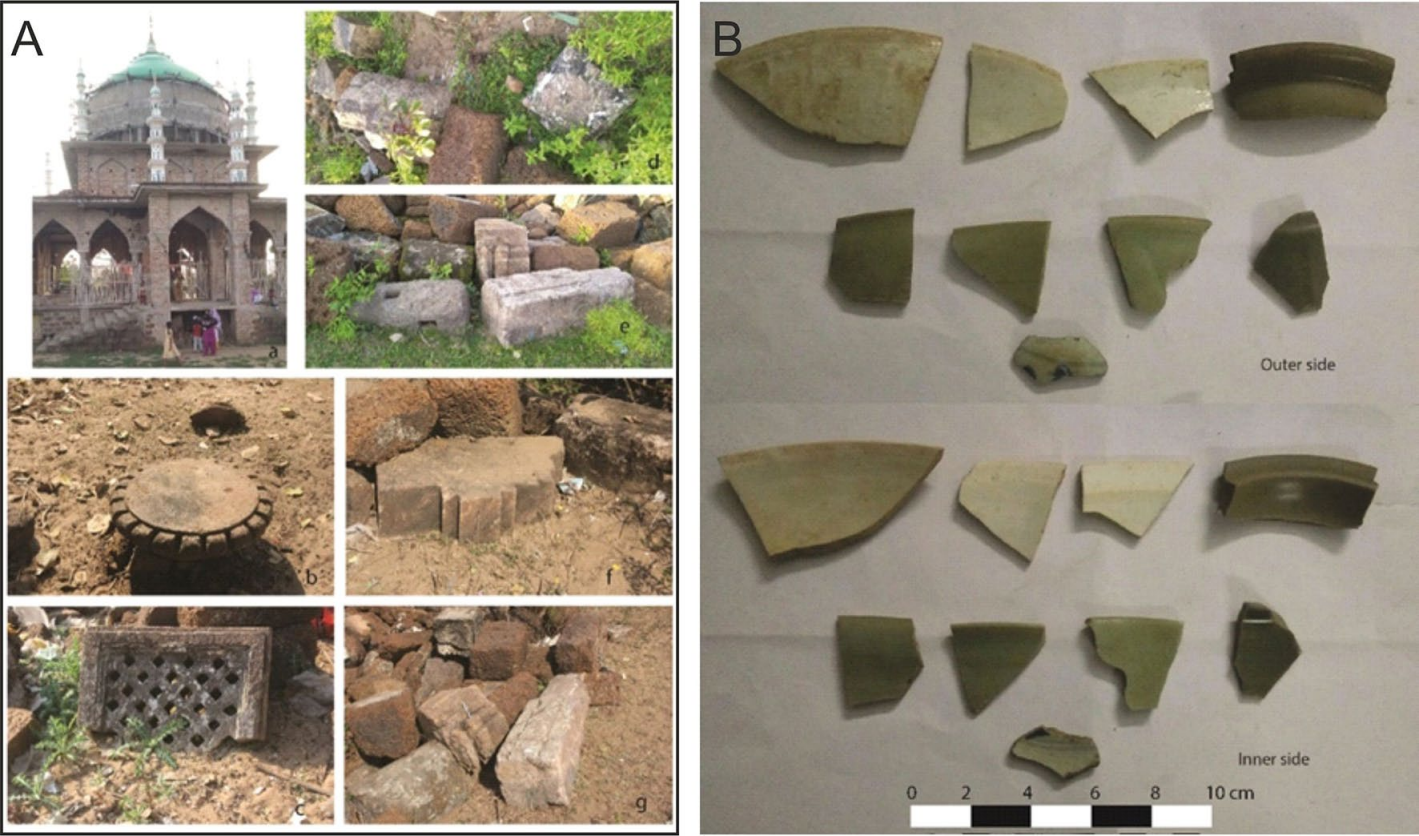
Architectural remains (**A**) and Chinese pottery found at Manikapatna, along with the stone anchor (**B**).

During the 2018–2019 exploration adjacent to the mosque of Manikapatna, a broken Indo-Arabian (Fig. 3) stone anchor was found, which was piled up along with fragments of *amalaka* (notched stone disk), perforated window pieces, pillar segments, dressed stone blocks. During the digging of the foundation for the construction of a new mausoleum these remains were recovered, which apparently belonged to a temple (Fig. 4A). In addition, Chinese ceramic sherds (Fig. 4B) of the 13th to 14th century CE were also recovered from the site, which were produced in Fujian and Zhejiang Provinces of China (*personal comm: Ran Zhang*).

## Sample and methods

The Indo-Arabian stone anchor of Manikapatna was broken when discovered (Fig. 3A). However, both the lower holes were intact and as expected from such anchors they were proportionately of different sizes, with one hole being filled with sand. The upper portion of the anchor, including the top hole for tying the rope, was missing—the likely outline of which is sketched for illustration purpose in Fig. 3B. From the appearance of the surface of the anchor, although had been neatly chiseled, the vesicular nature of the rock is obvious. The rock shows basaltic texture with small, white plagioclase crystals visible in the fine grained mafic/dark groundmass. Some of the vesicles are filled with secondary minerals.

**Figure 5:**
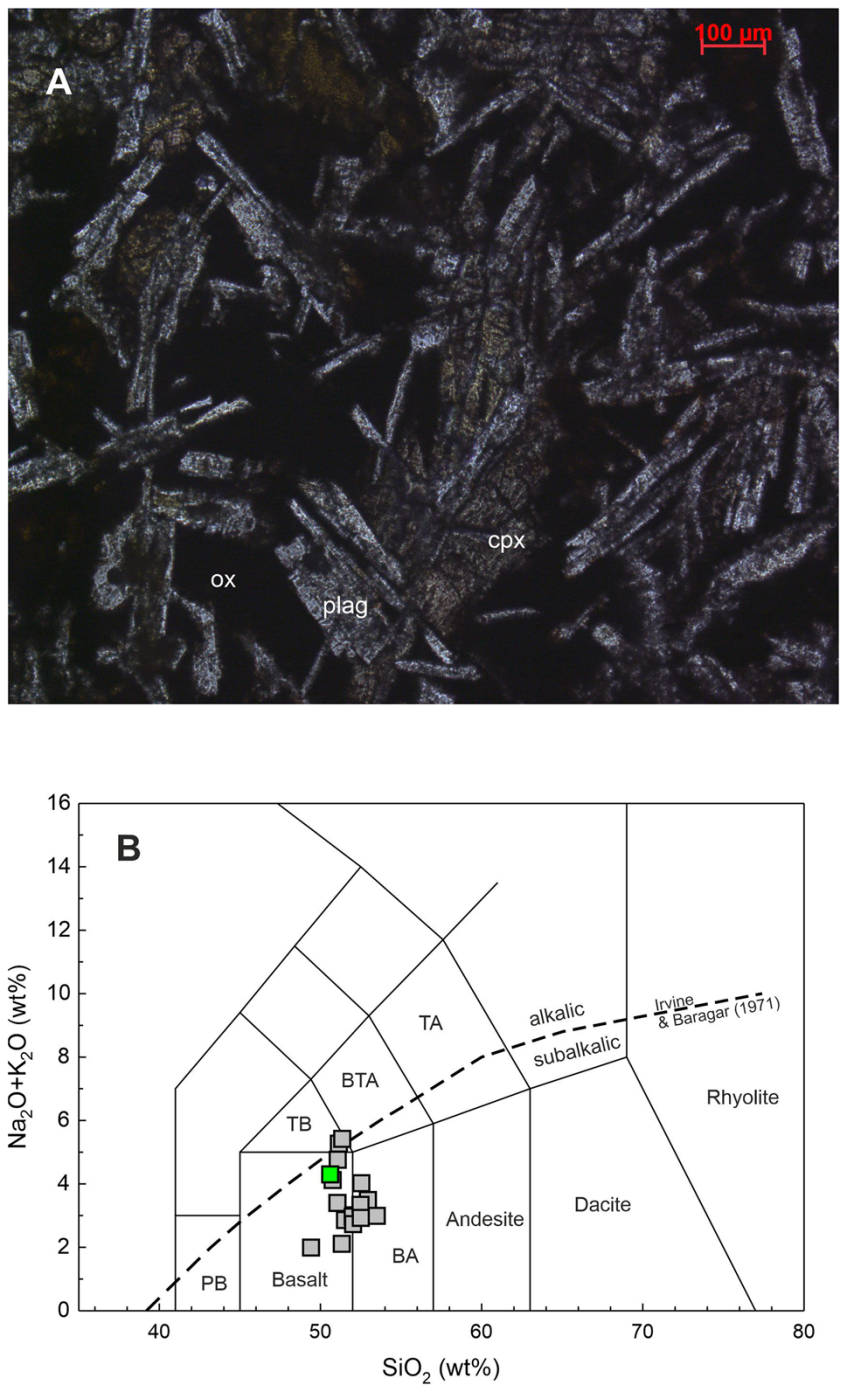
(**A**) Photomicrograph of a thin section of the sample chip from the Chilika Anchor showing plagioclase laths (plag), clino pyroxene (cpx) and dark iron oxides (ox). The texture seen is typical of a basaltic lava; (**B**) Anchor basalt datum (green square) plotted on a total alkali silica (TAS) diagram (Le Bas et al., 1986)^14^. The Boundary line between the alkalic and subalkalic field is by Irvine and Baragar (1971)^15^. Also plotted are data for lava flows of Palitana, Gujarat, India (data source: Sheth et al., 2013)^16^.

A couple of thin sections of the anchor rock were prepared and petrography was done using a polarizing microscope. Minerals in the rock were identified using their optical properties (Fig. 5A). Major element contents were analyzed on a pressed powder pellet of the sample by X-ray fluorescence (XRF) spectroscopy at the Physical Research Laboratory (PRL) using the Rigaku make Supermini200 machine and following the methods of Norrish and Chapell^13^ (1977). Multiple international rock standards were used for calibration and the BHVO-2 international basalt standard was used for accuracy check.

For elemental analyses, the powdered sample was digested in HF-HNO_3_ acid mixture and the solution was diluted in 2% HNO_3_. Measurements were done on Thermo Fisher make Element XR HR-ICPMS and instrumental drift was corrected using internal standards of Ga, In and Bi. Reproducibility was better than 1% (2σ) for rare earth elements, and 2% (2σ) for other trace elements based on repeated analyses of the BHVO-2. For Sr and Nd isotopic ratio analyses the sample powder was dissolved using the standard HF-HNO_3_-HCl dissolution protocol for silicates. Pure Sr and Nd were extracted by means of conventional cation exchange liquid chromatography with AG® 50W-X8 resin from Biorad and Ln-spec resin from Eichrom®, respectively, and using dilute HCl as elutant. ^87^Sr/^86^Sr and ^143^Nd/^144^Nd were measured on a Thermo Fisher make Triton Plus TIMS in static mode. Isotopic ratios were corrected for machine induced mass fractionation using internal constant ratios of 0.1194 and 0.7219, respectively for ^86^Sr/^88^Sr and ^146^Nd/^144^Nd and an exponential fractionation law. The measured values of ^87^Sr/^86^Sr of NBS 987 and ^143^Nd/^144^Nd of JNdi-1 were 0.710250 ± 0.000008 (2σ, n = 20) and 0.512104 ± 0.000004 (2σ, n = 20), respectively.

## Results

**Figure 6:**
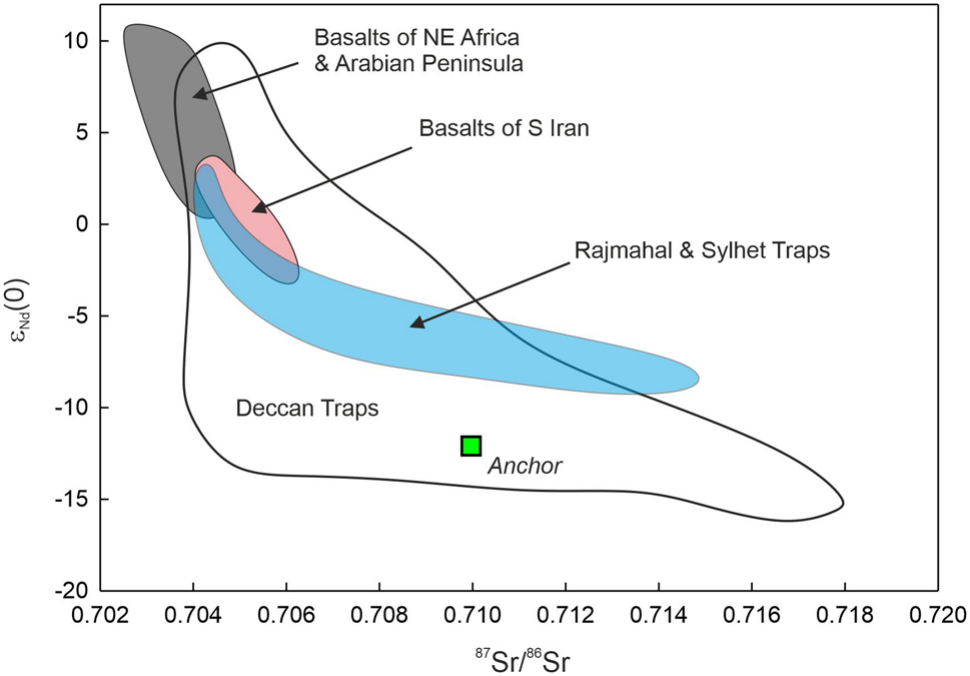
ε_Nd_(0) versus ^87^Sr/^86^Sr of the Chilika Anchor rock compared with the same for the Deccan Traps (Basu et al., 2020)^17^, basalts of north-eastern Africa and Arabian peninsula (Ethiopia, Kenya, Saudi Arabia and Yemen; Kieffer et al., 2004^18^ and references therein), basalts of southern Iran (Yeganehfar et al., 2013)^19^, and basaltic flows of Rajmahal and Sylhet Traps^20^.

**Figure 7:**
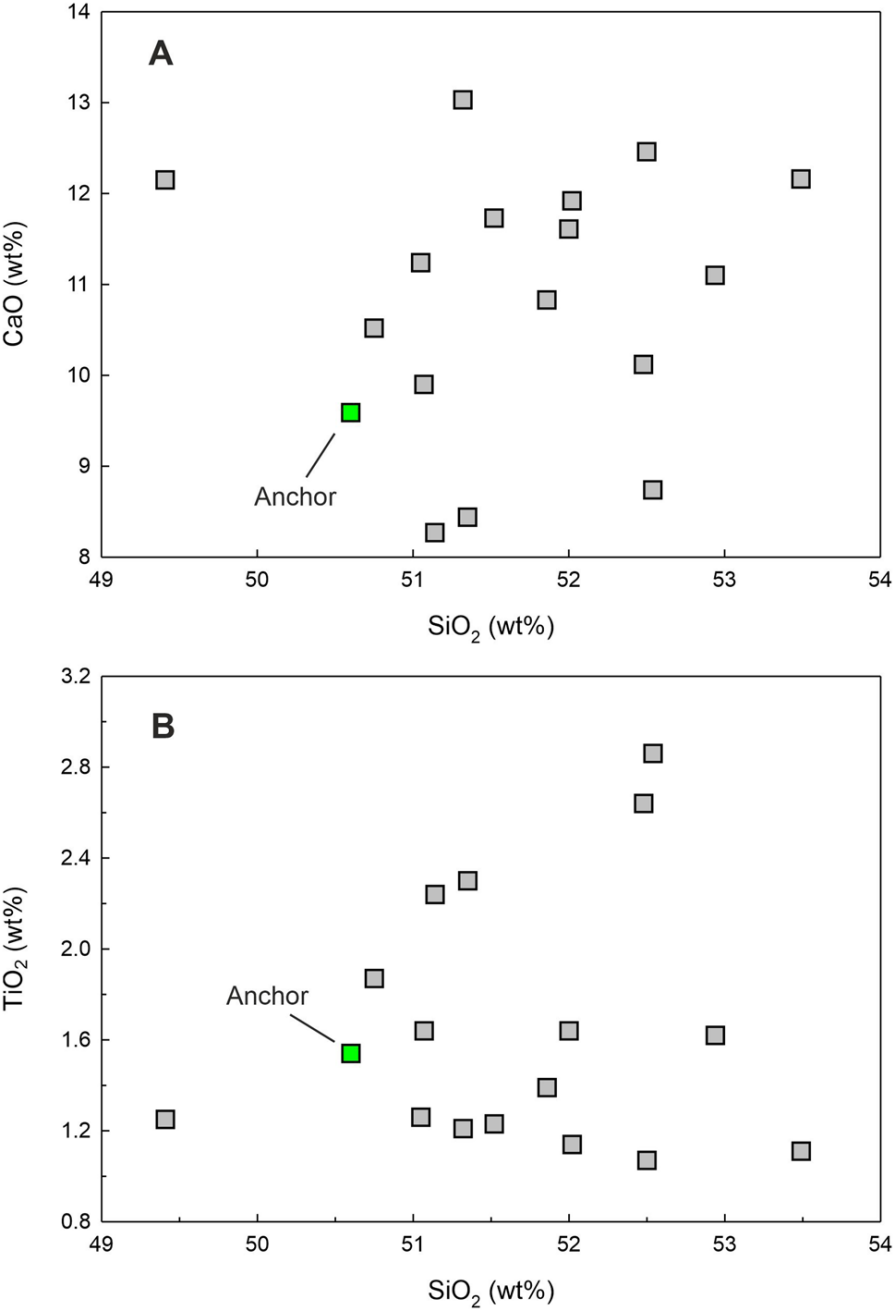
(**A**) CaO versus SiO_2_ and (**B**) TiO_2_ versus SiO_2_ for the Chilika Anchor rock compared with the data for Palitana lava flows (data source: Sheth et al., 2013)^16^.

**Figure 8:**
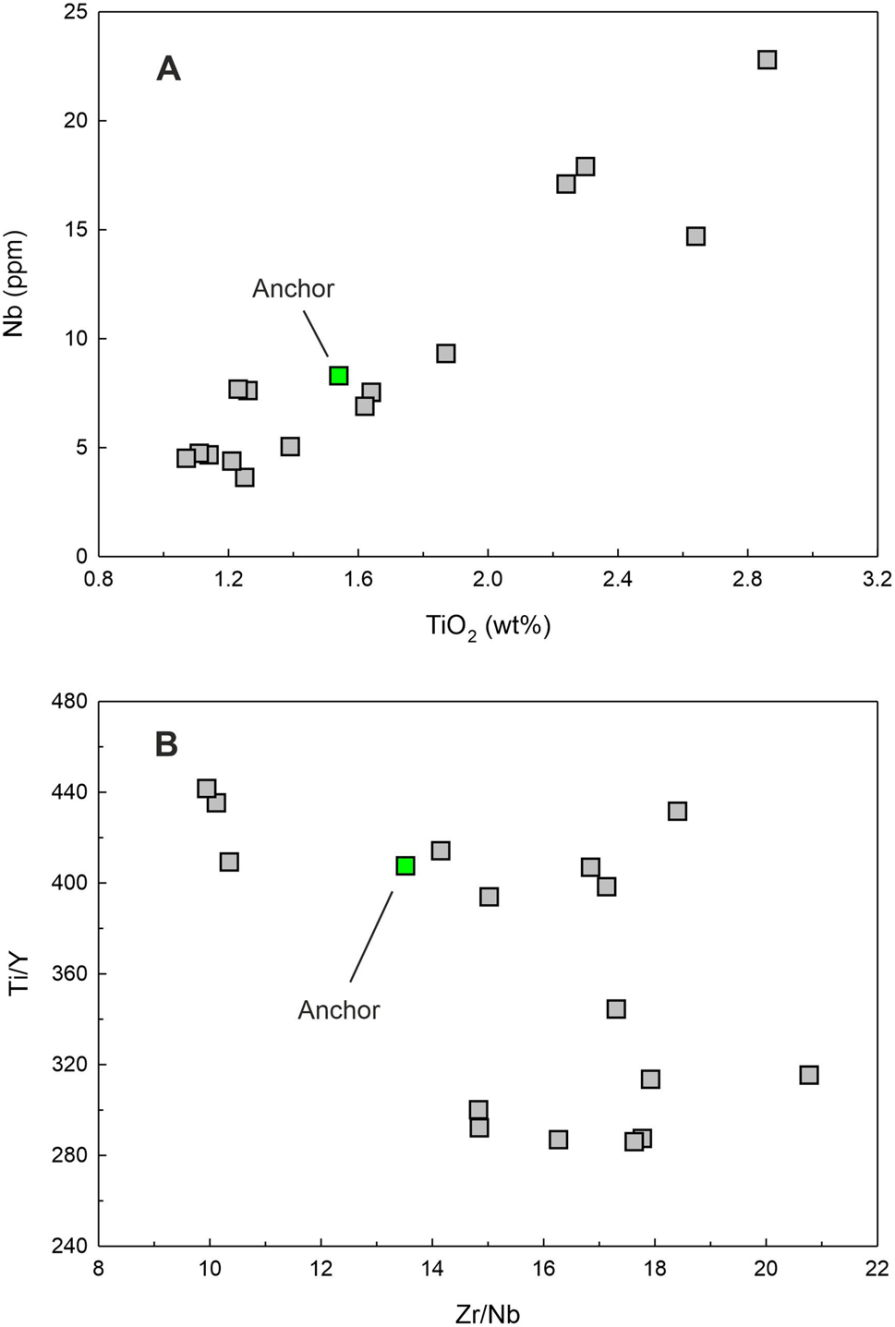
Cross plots of Nb vs. TiO_2_ (**A**) and Ti/Y vs. Zr/Nb (**B**) for the Chilika Anchor rock compared with the data for Palitana lava flows (data source: Sheth et al., 2013)^16^.

**Figure 9:**
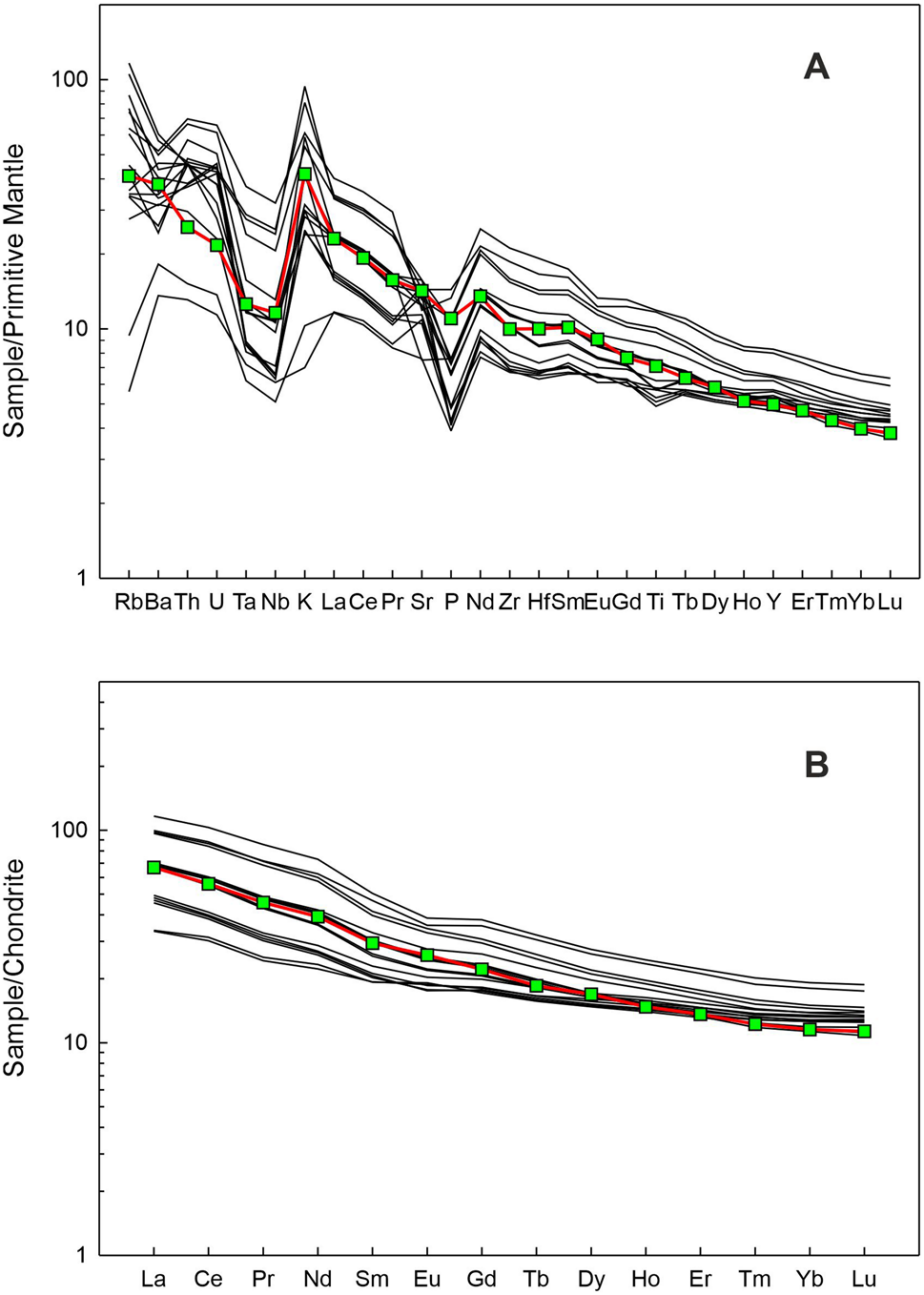
(**A**) Primitive Mantle normalized trace element pattern and (**B**) Chondrite normalized rare earth element pattern for the Chilika Anchor rock (green squares) compared with those for Palitana lava flows (data source: Sheth et al., 2013)^16^. Normalizing values are from Sun and McDonough (1989)^21^.

**Figure 10:**
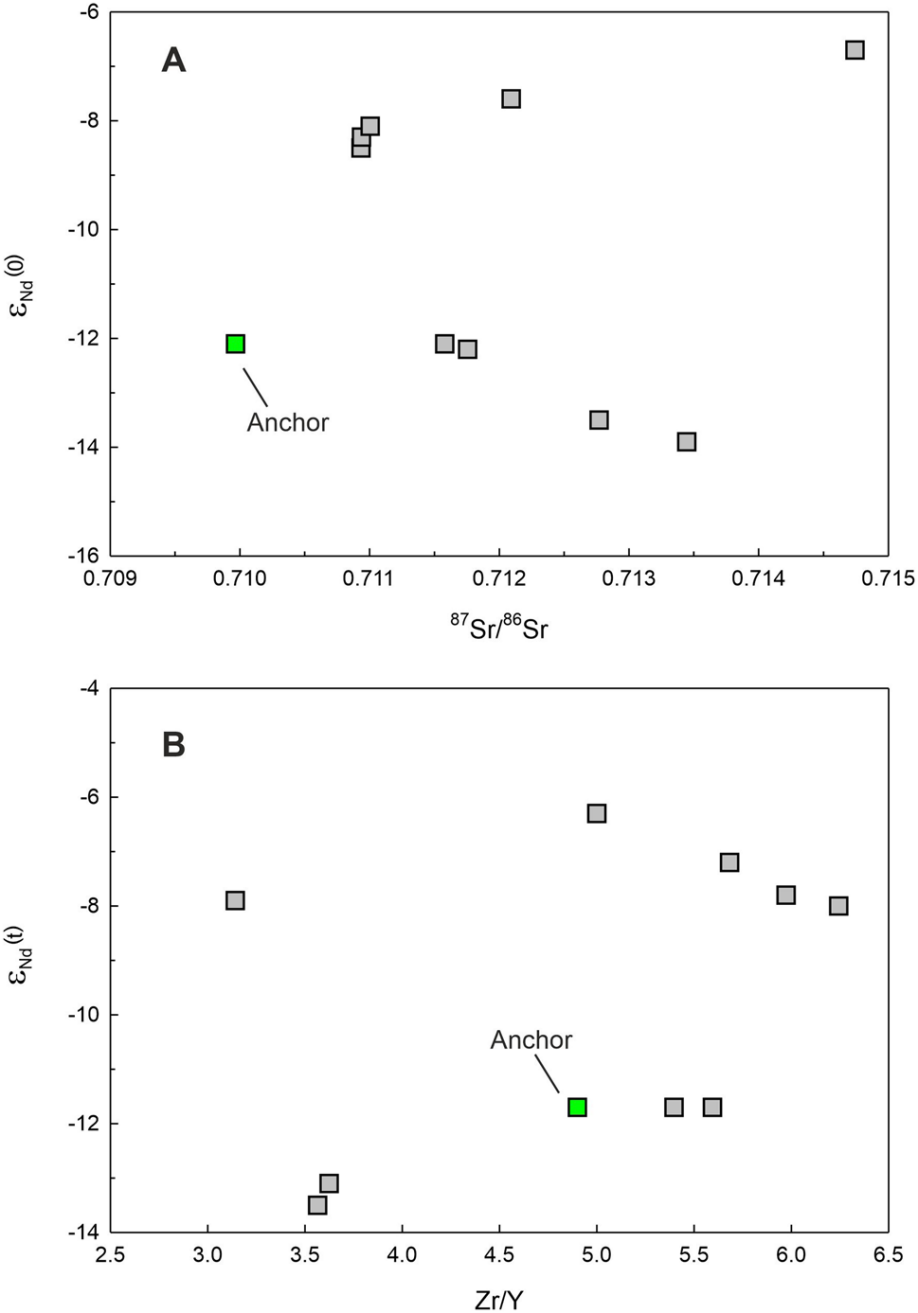
(**A**) ε_Nd_(0) versus ^87^Sr/^86^Sr and (B) ε_Nd_(t = 66 Ma) versus Zr/Y for the Chilika Anchor rock compared with the data for the Palitana lava flows (data source: Sheth et al., 2013)^16^.

The results of elemental content and isotopic ratio analyses of the anchor rock sample are presented in Table 2 and plotted in Figs. 5, 6, 7, 8, 9 and 10. The measured Nd isotopic ratio (^143^Nd/^144^Nd) is also presented as ε_Nd_(0) in Table 2 and Fig. 6, which is calculated as ε_Nd_(0) = [(^143^Nd/^144^Nd)_sample_/(^143^Nd/^144^Nd)_chondrite_ − 1] × 10^4^, where (^143^Nd/^144^Nd)_chondrite_ is taken as 0.512638. The ε_Nd_(t) in Table 2 and Fig. 10B is calculated using the above formula but with age corrected ratios at t = 66 Ma, the age of main eruptive event of the Deccan volcanism in India. The significance of this ratio shall be discussed later in the text. Figure 5A presents a photomicrograph of the thin section of the anchor rock in crossed (transmitted) polarized light. The light colored plagioclase crystals, the brown clino-pyroxene, altered groundmass, opaque minerals (Fe-oxides) and the overall texture are typical of a basalt.

**Table 2:** Geochemical and Isotopic compositions of the Manikapatna Stone Anchor

Composition	Data (wt%)
SiO_2_	50.6
TiO_2_	1.54
Al_2_O_3_	12.99
Fe_2_O_3_^T^	12.48
MgO	9.51
MnO	0.15
CaO	9.59
Na_2_O	3.04
K_2_O	1.26
P2O_5_	0.24
Total	101.4

Composition	Data (ppm)
Cs	0.2
Rb	26.1
Ba	266.0
Th	2.2
U	0.5
Nb	8.3
Ta	0.5
La	15.8
Ce	34.2
Pb	6.0
Pr	4.3
Sr	300.0
Nd	18.3
Zr	111.9
Hf	3.1
Sm	4.5
Eu	1.5
Gd	4.6
Tb	0.7
Dy	4.3
Y	22.6
Ho	0.8
Er	2.3
Tm	0.3
Yb	2.0
Lu	0.3
^87^Sr/^86^Sr_meas_	0.709967
^143^Nd/^144^Nd_meas_	0.512020
ε_Nd_(0)	− 12.1
ε_Nd_(t = 66 Ma)	− 11.7

Major element oxides are in wt% and trace element contents are in ppm. Total iron (superscript T) is reported as ferric oxide. Reproducibility for major oxides is < 3 wt%, for all trace element contents < 2% except for REE for which it is < 1% at 2σ level, based on the repeated analysis of USGS rock standard BHVO-2. The average values for NBS987 and JNdi-1 analysed over a period of 4 years are ^87^Sr/^86^Sr = 0.71023 ± 0.00001 and ^143^Nd/^144^Nd = 0.512104 ± 0.000004 (± 0.1 in ε_Nd_ units) at the 2σ level of uncertainty.

The rock has 9.51 wt% of MgO, 12.99 wt% Al_2_O_3_ and 50.6 wt% SiO_2_ which are characteristic of a basalt. In the Total Alkali Silica (TAS) classification of volcanic rocks, the anchor rock plots well within the field defined for basalts (Fig. 5B). The Mg# of the basalt is 43.6 (Table 2). Various cross plots of major element oxide data and trace element contents and ratios are plotted in Figs. 7 and 8. Figure 9 presents Primitive Mantle (PM) and Chondrite normalized trace element and rare earth elements (REE) patterns for our sample. As can be seen in the figure, the anchor rock shows large ion lithophile element (LILE) enriched (Fig. 9A) and light REE enriched patterns (Fig. 9B), however, it also shows distinct negative anomalies for Nb and Ta, and a positive anomaly for K (Fig. 9A). A minor negative anomaly is also observed for P. Isotopic ratio data for the anchor rock sample are plotted in Figs. 6 and 10. The measured and initial (t = 66 Ma) Sr and Nd isotopic ratios of the rock are ^87^Sr/^86^Sr = 0.709967 and ^87^Sr/^86^Sr_i_ = 0.709731, and ^143^Nd/^144^Nd_i_ = 0.512020 (ε_Nd_(0) = − 12.1) and ^143^Nd/^144^Nd_i_ = 0.511956 (ε_Nd_(t) = − 11.7), respectively.

## Discussion

### Nature of the anchor rock

The Manikapatna anchor rock is a basalt, as inferred from its mineralogy, texture and major element chemistry (Fig. 5). It has not undergone much alteration or metamorphism, and still has unfilled vesicles preserved on its surface (Fig. 3A), which suggests that the anchor must have come from a (geologically) young lava flow. Compositionally, the rock can be classified, based on TAS diagram, as a subalkalic basalt (Fig. 5B). The sample shows LILE and LREE enriched patterns (Fig. 9), which are suggestive of derivation from a LILE/LREE enriched mantle source; an Ocean Island Basalt (OIB) mantle source or a metasomatized continental lithospheric mantle source. However, the negative anomalies observed for Nb and Ta and positive anomaly for K suggest the involvement of continental crust as a contaminant. Derivation through a small degree of partial melting of an LREE depleted asthenospheric mantle source is unlikely as such a mechanism cannot explain the above anomalies. Derivation from an Island Arc type of mantle is also ruled out because the anchor rock lacks the typical subduction induced positive anomalies of Sr and Pb in PM/N-MORB normalized plots^22^ (Fig. 9A). The measured ^87^Sr/^86^Sr (0.709967) is much higher, and ε_Nd_ (− 12.1) is much lower than those expected for an uncontaminated young (< 120 Ma) mantle derived basaltic magma. These data hint at presence of significant amount of radiogenic Sr and non-radiogenic Nd in the parental melt that crystallized to produce the source rock of the anchor. Such chemical affinity is usually attributed to continental crustal material, which in turn suggests that the parental magma was contaminated by the crust through which it had erupted.

### Provenance of the anchor rock

The Indo-Arabian and other types of stone anchors have been recorded from maritime archaeological explorations in Dwarka, Bet Dwarka, Somnath, Miyani and Visawada of Gujarat, western India^23,24^. Besides, Indo-Arabian stone anchors have also been found during an inland survey of Aramda, Gopnath, Hatab, Ghogha and Mithi Virdi off the Gujarat coast^25^. Maritime explorations off Tamil Nadu^26^, east coast of India and Maharashtra^27–29^, Goa^30^, Kerala^31^, and Lakshadweep^32^ have also discovered Indo-Arabian stone anchors. More of these Indo-Arabian type stone anchors have been retrieved from the west coast of India compared to the east coast. A large number of these anchors, including the one that is the subject of the current study, have been found in localities adjacent to mosques, which possibly attest to their links to Arab (Islamic) mariners.

The determination of provenance of the anchor rocks is closely linked to the understanding of the trading routes in the ancient world. Since the typology of the anchors and the compositions of the rocks are so diverse that it is not yet fully deciphered whether most of them originated from local rock formations. Besides, very few studies had employed two of the most effective tools, the geochemical and isotopic methods, to address this issue. Earlier studies on Indo-Arabian anchors discovered in India have suggested their origin primarily from Gujarat region^12,33^. However, considering that similar rock formations are found elsewhere in India and in Arabian/Iranian territories, which had maritime contact with ancient India, it is imperative that more robust fingerprinting techniques such as (isotope) geochemistry is used for the purpose.

**Figure 11:**
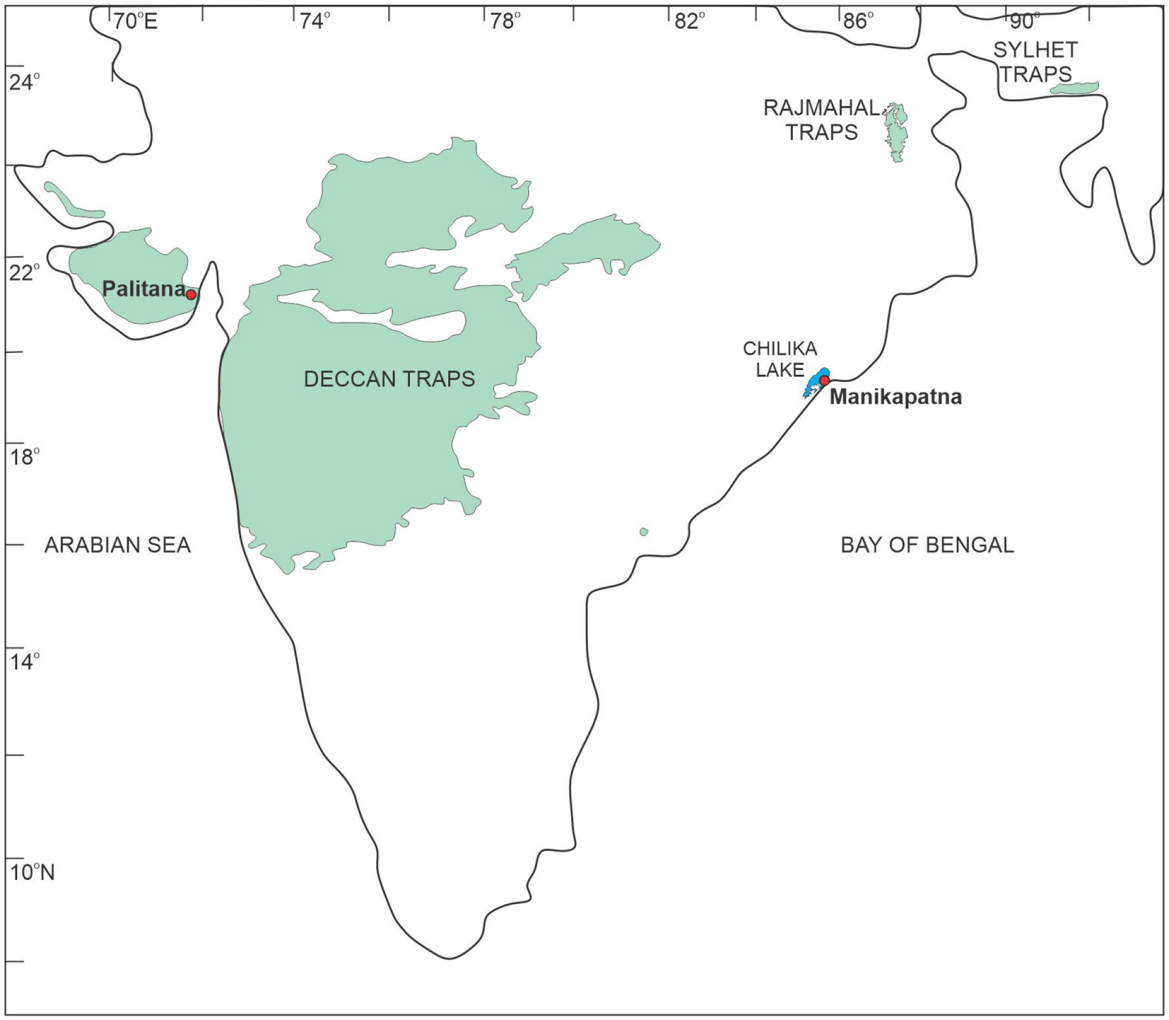
Map of peninsular India showing distribution of the Youngest (Cretaceous) Basaltic Volcanism in India: ~ 116 Ma Rajmahal-Sylhet Traps^35^; ~ 66 Ma Deccan Traps^36^. Locations of the discovery site Manikapatna and the source rock locality, Palitana in Gujarat, are marked.

The local rock formations around the Chilika lake, on which bank Manikapatna is located, are Proterozoic anorthosites, charnokites/migmatites or khondalites. No younger basaltic lavas are known from the state of Odisha. Since the Manikapatna anchor rock is inferred to have been cut out of a young basaltic lava flow, the likely candidate for its provenance in India is the ~ 66-million-year-old Deccan Traps (Fig. 11), located in the close vicinity of the western Indian coastline. The other possible source of rocks theoretically should lie in the Arabian region that has a rich history of Medieval maritime trading. These could have been the younger basalts of northeastern Africa, the Arabian Peninsula or southern Iran^18,19,34^. However, a simple comparison of the measured Sr and Nd isotopic compositions of the anchor rock with those of the basalts of the Arabian region indicates that the anchor definitely did not come to these volcanic rock formations (Fig. 6). On similar arguments, it can also be ruled out that the anchor rock did not come from the lava flows of the ~ 116-million-year-old Rajmahal or Sylhet Traps of eastern India (Fig. 11). Isotopic fingerprinting clearly suggests that the anchor was cut out from a basaltic lava flow of the Deccan Traps (Fig. 6). Therefore, the logical next step is to establish the exact locality of the lava flow in the Deccan volcanic province (DVP), a continental flood basalt province that covers almost one third of the area of western India (Fig. 11).

As discussed in the previous section, our anchor rock is a subalkalic basalt with low TiO_2_ and Nb (Fig. 8A) and such basaltic flows of Deccan Traps are generally observed in the Saurashtra region of Gujarat^16^, western India (Fig. 11), which is also home to the largest number of Indo-Arabian anchor finds^33^ (Fig. 1). Therefore, it is reasonable to presume that our anchor, discovered at Manikapatna, Odisha (Fig. 1), had originated from this region of Gujarat (Fig. 11). Of all the studied basaltic lava flows of the Saurashtra region, the flows of Palitana (Fig. 11) have the closest geochemical resemblance with the Manikapatna anchor (Figs. 5B, 7, 8, 9, 10). We make use of several major element oxides and trace element ratios that are conventionally used for (chemo)stratigraphic correlation of lava flows in the DVP and find that the parent lava flow of the anchor most likely is one of the many basaltic lava flows of Palitana (Figs. 5B, 7, 8)^16^. There are remarkable similarities between the PM-normalized trace element pattern and the Chondrite-normalized REE pattern of the anchor rock with those of the Deccan lava flows of Palitana (Figs. 9, 11), in fact, the anchor rock data fall well within the variabilities seen in the Palitana lava flows. Based on the isotopic source fingerprinting exercise we make the following inference: whereas the ^87^Sr/^86^Sr of the anchor is different from the known range of ^87^Sr/^86^Sr observed in Palitana lavas, its ε_Nd_ composition falls well within the observed range (Fig. 10A). The ^87^Sr/^86^Sr of the anchor represents a less altered or a less contaminated lava flow, or have not yet been sampled for such isotopic studies. Further confirmation of the provenance comes from the ε_Nd_ (t = 66 Ma) vs. Zr/Y plot (Fig. 10B) which clearly shows that the source rock of the anchor and some Palitana lavas have had similar genetic history.

### Implications

The chemical and isotopic fingerprinting of the stone anchor of Manikapatna ascertains that the provenance of the rock was Palitana in the Bhavnagar district Saurashtra region of Gujarat (Fig. 11). A mariner either from the Arabian Peninsula of nearby states or from Gujarat or Odisha might have brought this particular stone anchor from (the east coast of) Gujarat to Manikapatna, which confirms the existence of internal contacts between Gujarat and Odisha during the Medieval period. Maritime contacts between Manikapatna and Gujarat during ancient times are well known from historical records, for instance, Manikapatna is mentioned in the sixteenth and seventeenth century maps^37^ and referred to in the Gujarati sea manuals of the eighteenth century^10^. Further, Abul Fazal (1551–1602 CE) mentioned Manikapatna as a port where salt taxes were collected^38^. Bowery (1669–1679) mentioned the maritime activities of Manikapatna port, which was located along the coast of Gingelly^39^ and engaged in the transportation of food grains and calicoes^40^. Similarly, a Persian inscription^41^ found at the mosque of Manikapatna states that Mohammed Kamal constructed the mosque at Manikapatna in 1193 Hizra (1779 CE), during the time of Shah Alam II, the Mughal Emperor. Among several Muslim pir tombs of Odisha, Manikapatna^42^ has one. It could be assumed that either an Arab or Muslim mariner might have brought the stone anchor to Manikapatna and kept it at the mosque of Manikapatna as a symbol of passion because such examples have been reported earlier from both the east and west coasts of India. The exact reason behind the abandonment of the anchor remains unknown.

Ample evidence exists about strong maritime trading between the port towns in the Gulf of Cambay such as Gogha, Gandhar, Broach, Rander, Surat and Gandevi of Gujarat, and the ports along the coasts of Odisha and Bengal, and many Southeast Asian countries during the rule of the Mogul emperor Akbar (1556–1605) and for more than a century afterwards^43^. This trade finds mentioned in “Ain-i-Akbari” or the "Administration of Akbar". The traders of the Cambay region primarily dealt with the textiles of Odisha^44^. The cotton textiles of Odisha, Bengal, Gujarat and Tamil areas were also supplied to the Southeast Asian markets since at least the fifteenth century^45^. Because such contacts existed, the Indo-Arabian stone anchor of Manikapatna could likely have been brought by some traders of the Ghogha region of Gujarat. Since the distance between Ghogha (seaport; Fig. 1) and Palitana (anchor source; Fig. 11) is less than 60 km, we speculate that the anchor was made at Ghogha after transporting the rock from Palitana. Such a scenario is supported by the discovery of many intact, broken and uncompleted stone anchors at Ghogha^46^, which suggests that Ghogha was also an anchor manufacturing centre, besides being an active port of the Medieval period.

## Conclusions

The chemical and isotopic compositions of the stone anchor of Manikapatna on the Chilika Lake, Odisha, closely resemble that of the subalkalic basalt lava flows of the DVP near Palitana in the Bhavnagar district Saurashtra region of Gujarat. The absence of such young basalt in Odisha suggests that the stone anchor was transported from the Saurashtra coast. The provenance study of the stone anchor proves direct maritime contacts between Odisha and Gujarat during the Medieval Period, and this could be further corroborated with the references made in the historical records. Further explorations along the Chilika coast may yield more such stone anchors, which would provide a further understanding of the regional and external interactions of India during ancient times.

Despite the advancement of tools and technology in the field of shipping, and shipbuilding, i.e., wood to steel and sail to steam and stellar to satellite navigation, traditional fishermen preferred the Indo-Arabian stone anchors until very recently, suggesting continuity of ancient traditions/usages. Thus, it is essential to study and understand the indigenous knowledge of shipping and maritime Archaeology.

## Data Availability

All raw data used in the manuscript are presented in the Tables 1 and 2.
